# Hepatoprotective Effect of *Cuscuta campestris* Yunck. Whole Plant on Carbon Tetrachloride Induced Chronic Liver Injury in Mice

**DOI:** 10.3390/ijms17122056

**Published:** 2016-12-07

**Authors:** Wen-Huang Peng, Yi-Wen Chen, Meng-Shiou Lee, Wen-Te Chang, Jen-Chieh Tsai, Ying-Chih Lin, Ming-Kuem Lin

**Affiliations:** 1 Department of Chinese Pharmaceutical Sciences and Chinese Medicine Resources, College of Biopharmaceutical and Food Sciences, China Medical University, 91 Hsueh-Shih Road, Taichung 40402, Taiwan; whpeng@mail.cmu.edu.tw (W.-H.P.); kathy@hsbf.com.tw (Y.-W.C.); leemengshiou@mail.cmu.edu.tw (M.-S.L.); wtchang@mail.cmu.edu.tw (W.-T.C.); 2 Department of Health and Nutrition Biotechnology, College of Health Science, Asia University, 500 Liufeng Rd., Wufeng, Taichung 41354, Taiwan; jenchieh@mail.dyu.edu.tw; 3 Department of Optometry, Jen-Teh Junior College of Medicine, Nursing and Management, 79-9 Sha-Luen Hu Xi-Zhou Li Hou-Loung Town, Miaoli 356, Taiwan; u462013@yahoo.com.tw; 4 Graduate Institute of Biotechnology, National Chung Hsing University, 145 Xingda Rd., South Dist., Taichung 402, Taiwan

**Keywords:** *Cuscuta campestris*, hepatoprotective effect, carbon tetrachloride, fibrosis, antioxidant effect

## Abstract

Cuscuta seeds and whole plant have been used to nourish the liver and kidney. This study was aimed to investigate the hepatoprotective activity of the ethanol extract of *Cuscuta campestris* Yunck. whole plant (CC_EtOH_). The hepatoprotective effect of CC_EtOH_ (20, 100 and 500 mg/kg) was evaluated on carbon tetrachloride (CCl_4_)-induced chronic liver injury. Serum alanine aminotransferase, aspartate aminotransferase, triglyceride and cholesterol were measured and the fibrosis was histologically examined. CC_EtOH_ exhibited a significant inhibition of the increase of serum alanine aminotransferase, aspartate aminotransferase, triglyceride and cholesterol. Histological analyses showed that fibrosis of liver induced by CCl_4_ were significantly reduced by CC_EtOH_. In addition, 20, 100 and 500 mg/kg of the extract decreased the level of malondialdehyde (MDA) and enhanced the activities of anti-oxidative enzymes including superoxide dismutase (SOD), glutathione peroxidase (GPx) and glutathione reductase (GRd) in the liver. We demonstrate that the hepatoprotective mechanisms of CC_EtOH_ were likely to be associated to the decrease in MDA level by increasing the activities of antioxidant enzymes such as SOD, GPx and GRd. In addition, our findings provide evidence that *C. campestris* Yunck. whole plant possesses a hepatoprotective activity to ameliorate chronic liver injury.

## 1. Introduction

Cuscuta seed, Cuscutae Semen or Tu-Si-Zi, which has been widely used to nourish the liver and kidney, mainly refer to the seeds of *Cuscuta chinensis* Lam. Some phytochemical and pharmacological studies have reported the beneficial activities of Cuscuta seeds [1]. For example, Cuscuta seeds have activities to improve defective kidneys [2], prevent liver against damage [3] and alleviate inflammation/pain [4]. Crude polysaccharides from Cuscuta seeds have an immunostimulating activity [5]. Alongside with the use of the seeds, the Cuscuta whole plant has also been recorded in the famous book “Shen Nong’s Herbal” and some ancient medicinal books to treat dermatosis. In addition, it has been used as a folk medicine to treat adiposity or as a substitute for the Cuscuta seeds. However, no pharmaceutical study has been reported yet. In this study, *C. campestris* Yunck. whole plant was locally collected and used for the first time to examine its pharmaceutical activity.

Liver tissue injury can be caused by the ingestion of chemicals or drugs or by infection through virus infiltration [6]. Among them, carbon tetrachloride (CCl_4_) is commonly used to study hepatotoxicity in animal models [7,8]. CCl_4_ can be metabolized into the highly reactive trichloromethyl radical [9] and then trigger lipid peroxidation [10]. Therefore, blocking the lipid peroxidation can protect liver against CCl_4_-induced injury [11,12]. In this study, the hepatoprotective activity of the ethanol extract of *C. campestris* whole plant (CC_EtOH_) was investigated on CCl_4_-induced chronic liver injury in mice. Once liver damage has occurred, liver marker enzymes (alanine aminotransferase (ALT), and aspartate aminotransferase (AST)) and lipid profile (total triglyceride and cholesterol) will be increased [13]. Therefore, the levels of serum ALT, AST, cholesterol and triglyceride were measured in this study. In addition, liver biopsies were performed for examining the pathological changes. To elucidate the underlying mechanism of the hepatoprotective activity, the levels of malondialdehyde (MDA) and the activities of anti-oxidative enzymes (superoxide dismutase (SOD), glutathione peroxidase (GPx), and glutathione reductase (GRd)) in liver were also measured. Silymarin was examined as the positive control as it is a promising agent for liver protection. Our findings in this study provide evidence that *C. campestris* Yunck. whole plant possesses a hepatoprotective activity and the underlying mechanism is likely to be associated to the increase of the anti-oxidation by increasing the activities of antioxidant enzymes such as SOD, GPx and GRd.

## 2. Results and Discussion

### 2.1. Effect of CC_EtOH_ on CCl_4_-Induced Hepatotoxicity

**Figure 1:**
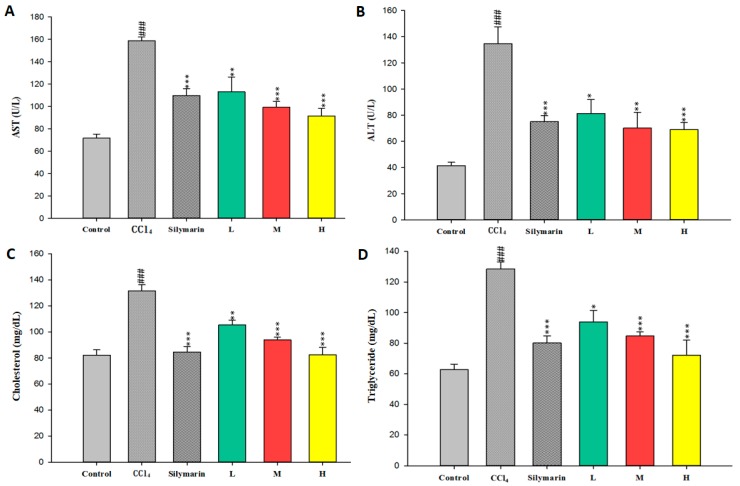
The effects of silymarin and the ethanol extracts of *Cuscuta campestris* whole plant at low (L), middle (M) and high (H) concentrations (20, 100 and 500 mg/kg, respectively) on serum: aspartate aminotransferase (AST) (**A**); and alanine aminotransferase (ALT) (**B**) activities; and cholesterol (**C**); and triglyceride (**D**) levels in mice treated with CCl_4_. Values are mean ± SEM (*n* = 10). ^#^ indicates significant difference from the control group (^###^ *p* < 0.001). * indicates significant difference from the CCl_4_ group (* *p* < 0.05, ** *p* < 0.01 and *** *p* < 0.001).

First of all, the hepatotoxicity effect of CCl_4_ and the protective effect of CC_EtOH_ were examined using the serum of CCl_4_-induced mice. As shown in Figure 1, the CCl_4_ group exhibited significant increases of serum ALT, AST, triglyceride and cholesterol. However, these increases were obviously inhibited by treatment with CC_EtOH_ (20, 100 and 500 mg/kg) and silymarin (200 mg/kg). In addition, the inhibitions were in a dose-dependent manner. These results clearly suggested that CC_EtOH_ possess protective properties against CCl_4_-induced liver injury.

### 2.2. Histological Analyses

**Figure 2:**
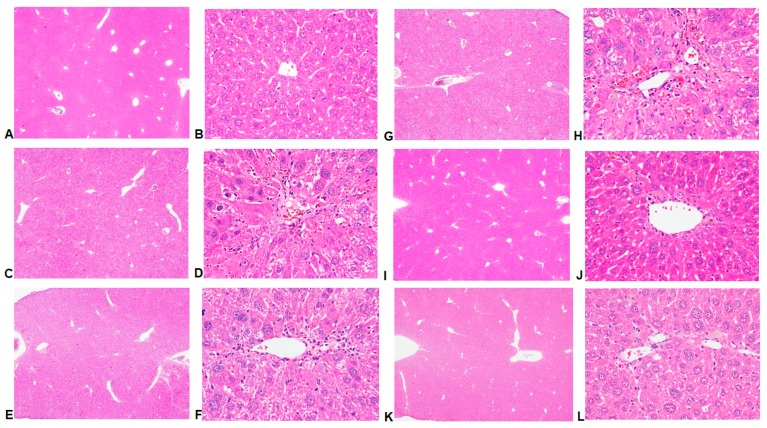
Hepatic histological analyses of the effects of silymarin and the ethanol extracts of *Cuscuta campestris* whole plant (CC_EtOH_) on CCl_4_-induced liver damage in mice using H&E staining (40× (**A**,**C**,**E**,**G**,**I**,**K**) and 200× (**B**,**D**,**F**,**H**,**J**,**L**) magnification): (**A**,**B**) control group; (**C**,**D**) animals treated with CCl_4_; (**E**,**F**) animals treated with silymarin (200 mg/kg) and CCl_4_; and (**G**–**L**) animals treated with CC_EtOH_ (20, 100 and 500 mg/kg) and CCl_4_, respectively.

**Figure 3:**
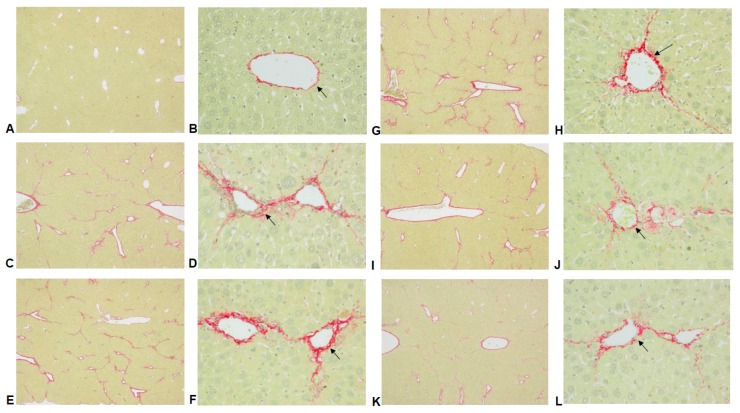
Hepatic histological analyses of the effects of silymarin and the ethanol extracts of *Cuscuta campestris* whole plant (CC_EtOH_) on CCl_4_-induced liver damage in mice using Sirius Red staining (40× (**A**,**C**,**E**,**G**,**I**,**K**) and 200× (**B**,**D**,**F**,**H**,**J**) magnification): (**A**,**B**) control group; (**C**,**D**) animals treated with CCl_4_; (**E**,**F**) animals treated with silymarin (200 mg/kg) and CCl_4_; and (**G**–**L**) animals treated with CC_EtOH_ (20, 100 and 500 mg/kg) and CCl_4_, respectively. Arrows indicate the fibrosis.

The results of hematoxylin and eosin histological analyses showed that CCl_4_ induced histological changes including increased hepatic cells cloudy swelling, cytoplasmic vacuolization, lymphocytes infiltration, hepatocellular and necrosis (Figure 2C,D) when compared to the control group (Figure 2A,B). The liver damages were reduced by treatment with CC_EtOH_ (20, 100 and 500 mg/kg) (Figure 2G–L). Since CCl_4_ induced fibrosis, Sirius Red staining was conducted and the score of liver fibrosis were examined. The results showed that the levels of inflammation and fibrosis are significantly decreased by treatment with CC_EtOH_ (100 and 500 mg/kg) (Figure 2 and Figure 3 and Table 1). Histological examinations showed that treatment with CC_EtOH_ significantly prevents CCl_4_-induced liver injury.

### 2.3. Effect of CC_EtoH_ on MDA Level

**Figure 4:**
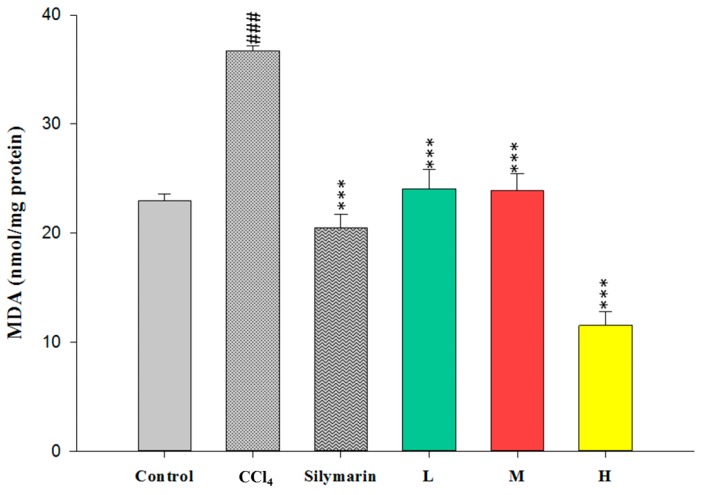
The effects of silymarin and the ethanol extracts of *Cuscuta campestris* whole plant on malondialdehyde (MDA) content in mice treated with CCl_4_. Values are mean ± SEM (*n* = 10). ^#^ indicates significant difference from the control group (^###^ *p* < 0.001). * indicates significant difference from the CCl_4_ group (*** *p* < 0.001).

As MDA level is usually used to elucidate the level of lipid peroxidation in liver, the effects of CC_EtOH_ on CCl_4_-induced MDA production were examined. As shown in Figure 4, the level of MDA in the CCl_4_ group was dramatically increased (*p* < 0.001) compared with the control group, however, the levels of MDA were significantly reduced by treatment with CC_EtOH_ (20, 100 and 500 mg/kg) (*p* < 0.001) and silymarin (200 mg/kg) (*p* < 0.001) compared with the CCl_4_ group. The results suggested that the CCl_4_-induced hepatic lipid peroxidation is reduced by CC_EtOH._

### 2.4. Effect of CC_EtOH_ on Antioxidant Enzymatic Activities

**Figure 5:**
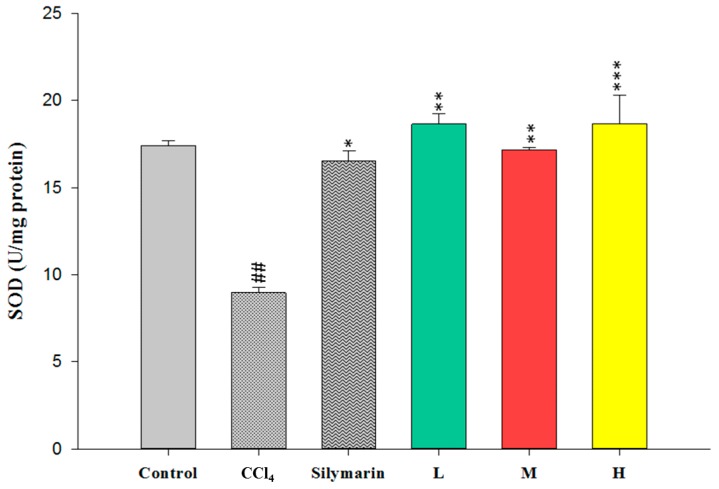
The effects of silymarin and the ethanol extracts of *Cuscuta campestris* whole plant on superoxide dismutase (SOD) activity in mice treated with CCl_4_. Values are mean ± SEM (*n* = 10). ^#^ indicates significant difference from the control group (^##^ *p* < 0.01). * indicates significant difference from the CCl_4_ group (* *p* < 0.05, ** *p* < 0.01 and *** *p* < 0.001).

**Figure 6:**
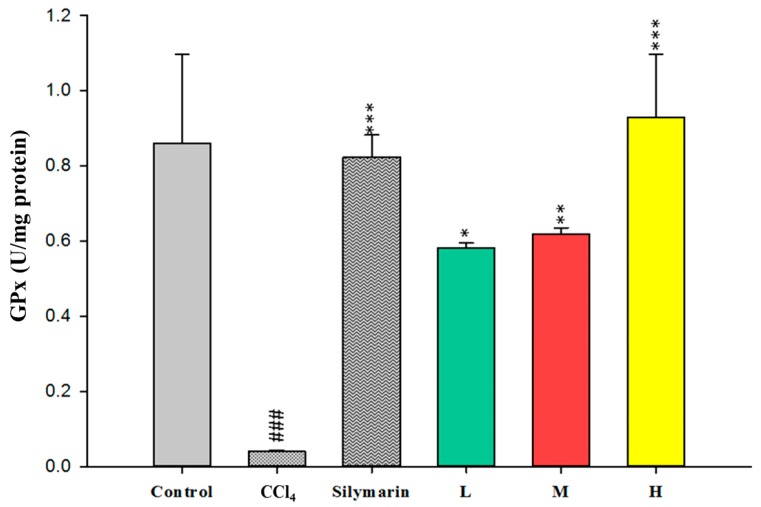
The effects of silymarin and the ethanol extracts of *Cuscuta campestris* whole plant on glutathione peroxidase (GPx) activity in mice treated with CCl_4_. Values are mean ± SEM (*n* = 10). ^#^ indicates significant difference from the control group (^###^ *p* < 0.001). * indicates significant difference from the CCl_4_ group (* *p* < 0.05, ** *p* < 0.01 and *** *p* < 0.001).

**Figure 7:**
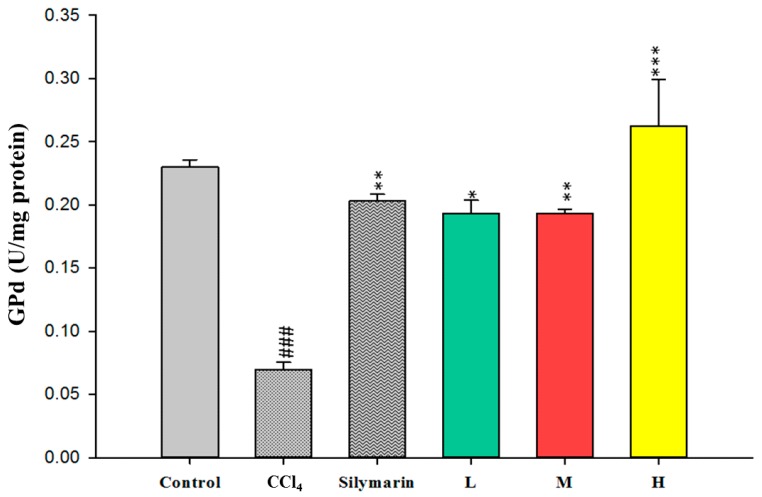
The effects of silymarin and the ethanol extracts of *Cuscuta campestris* whole plant on glutathione reductase (GRd) activity in mice treated with CCl_4_. Values are mean ± SEM (*n* = 10). ^#^ indicates significant difference from the control group (^###^ *p* < 0.001). * indicates significant difference from the CCl_4_ group (* *p* < 0.05, ** *p* < 0.01 and *** *p* < 0.001).

To evaluate the antioxidant effects of CC_EtOH_, SOD, GPx and GRd were measured in the liver. The activities of these hepatic enzymes in the CCl_4_ group were dramatically decreased compared with the control group (Figure 5, Figure 6 and Figure 7). However, treatment with CC_EtOH_ at the three doses and silymarin significantly increased the levels of SOD, GPx and GRd activities. The results suggested that the inhibitory effect of CCl_4_ on these hepatic enzymes was reversed by CC_EtOH_.

### 2.5. Phytochemical Analysis of CC_EtOH_

As shown in Figure S1, the HPLC chromatograms of CC_EtOH_ and the standards showed that peaks at the retention times of 17.5 and 24.5 min were hyperoside and quercetin, respectively. Two peaks showing at the retention times of 17.5 and 24.5 min were detected in the CC_EtOH_ chromatogram. The results revealed that hyperoside, quercetin and their glycosides are present in CC_EtOH_.

Cuscuta whole plant has also been used as a folk medicinal material to treat adiposity or as a substitute for the Cuscuta seeds, which have been widely used to nourish the liver and kidney in Chinese medicine. In this study, we demonstrated in the first time that the whole plant of *C. campestris* exhibits a hepatoprotective activity.

CCl_4_ has been commonly employed for the evaluation of hepatoprotective activity of different kinds of herbal extracts and drugs [8,15]. CCl_4_ is thought to be transformed into trichloromethyl radicals, which are hepatotoxic metabolites. These radicals are able to react with sulfhydryl groups of glutathione (GSH) and protein. In addition, they can trigger protein oxidation and lipid peroxidation, which result in hepatocellular damage [7,12,16]. In this study, CCl_4_ was used to induce chronic hepatic injury and our results revealed that the injury was significantly reduced by CC_EtOH_, clearly demonstrating that *Cuscuta campestris* possesses hepatoprotective activity.

Previous studies have shown that hepatic damage increases AST and ALT activities in the hepatocytes [16] and the levels of ALT, AST, triglyceride and cholesterol in serum are increased by administering CCl_4_ to mice [13,17,18]. In this study, serum ALT, AST, triglyceride and cholesterol levels were increased after CCl_4_ administration and these increases were all significantly decreased by treatment with CC_EtOH_ at three concentrations (Figure 1). In addition, histological analyses showed that hepatic cell injury induced by CCl_4_ was accompanied by fibrosis and such injury was attenuated by CC_EtOH_ (Figure 3). The quantitative histopathologic score of the fibrosis of hepatocytes showed that the fibrosis levels were significantly decreased by CC_EtOH_ (100 and 500 mg/kg; Table 1). These results indicated that CC_EtOH_ can prevent liver against fibrosis.

Lipid peroxidation has been shown to be an important cause of CCl_4_-induced liver injury [10]. Malondialdehyde (MDA) is the end product of the lipid peroxidation and thus commonly used as an indicator of the CCl_4_-induced liver injury [19]. In this study, the increased hepatic MDA levels induced by CCl_4_ were significantly decreased by treatment of CCl_4_ (Figure 4). Therefore, these results indicated that CCl_4_ can protect the liver against CCl_4_-induced injury through inhibiting MDA production. Superoxide dismutase (SOD), glutathione peroxidase (GPx) and glutathione reductase (GRd) are anti-oxidative enzymes which are easily inactivated by reactive oxygen species (ROS) and lipid peroxides which are caused by CCl_4_ [16]. In this study, the activities of SOD, GPx and GRd from the CCl_4_-induced injury livers were measured. The results showed that their activities were promoted by treatment with CC_EtOH_ (Figure 5, Figure 6 and Figure 7), suggesting that CC_EtOH_ is able to reduce ROS production by increasing hepatic anti-oxidative enzymes activities and thus prevent the development of CCl_4_-induced liver damage. To confirm the anti-oxidative activity of the extract used in this study, the catechin-equivalent phenolics and quercetin-equivalent flavonoid concentrations of the extract were examined and determined as 58.61 ± 0.8 and 15.032 ± 1.3 mg/g CC_EtOH_, respectively. In addition, 1,1-Diphenyl-2-picrylhydrazyl (DPPH) scavenging of the extract was examined. The catechin equivalent DPPH scavenging capability was determined as 25.19 ± 0.54 mg/g CC_EtOH_ and the IC_50_ of CC_EtOH_ for DPPH scavenging is approximately 1.71 mg/mL. These results support that CC_EtOH_ containing hyperoside and quercetin has a capability to increase the anti-oxidant systems in liver. Moreover, the inhibitory effect on MDA production was also likely due to the increase in SOD, GPx and GRd activities.

Phytochemical analyses by HPLC showed that the major compounds in CC_EtOH_ are hyperoside, quercetin and flavonoid glycosides (Figure S1). Although hyperoside and quercetin have been detected in both of seeds and whole plant of *C. campestris*, their amounts and the other flavonoids are different [4,20]. Hyperoside have been shown to increase the level of heme oxygenase-1, an important enzyme in antioxidant defense systems to reduce oxidative stress [21]. Quercetin has shown a high antioxidant activity by reducing the production of reactive oxygen species and nitric oxide [20,22]. The methanol extract of the seeds of *Cuscuta chinensis* containing hyperoside, quercetin and kaempferol have been reported to increase the activities of SOD, GPx and GRd in the liver [4]. Hyperoside and quercetin have been shown to exhibit hepatoprotective effect against CCl_4_-induced liver injury [23,24]. Therefore, hyperoside and quercetin can be the major active constituents in CC_EtOH_ which contribute to the hepatoprotective effect.

## 3. Materials and Methods

### 3.1. Plant Materials and Preparation of Plant Extract

*Cuscuta campestris* Yunck. grown on *Bidens pilosa* var. radiata was collected from Miaoli County, Taiwan. They were authenticated by Ming-Kuem Lin and Wen-Huang Peng in several aspects, including the morphology of its flowers and the chemical compositions of its seeds [20]. The whole plants of *C. campestris* Yunck. were dried in a circulating air oven, and then ground. The powder (1.05 kg) was extracted with 75% ethanol three times. The filtrates were collected and concentrated with a rotary evaporator under reduced pressure. The concentrated extract was then lyophilized and weighted. The yield ratio of CC_EtOH_ (91 g) was 8.7% (*w*/*w*). The extract was stored in −20 °C before the experiments.

### 3.2. Chemicals

Silymarin, quercetin and kampferol were purchased from Sigma-Aldrich Chemical Co. (Saint Louis, MO, USA). Carboxymethylcellulose (CMC) and carbon tetrachloride (CCl_4_) was purchased from Merck Co. (Munchen, Germany). CCl_4_ was dissolved into olive oil as a 40% (*v*/*v*) solution. All other reagents used were of analytical grades (Merck Co., Munchen, Germany).

### 3.3. Experimental Animals

ICR male mice (18–22 g) were purchased from BioLASCO Taiwan Co., Ltd. (Taipei, Taiwan). These mice were maintained in standard cages with a 12-h:12-h light-dark cycle, relative humidity 55% ± 5%, and 22 ± 1 °C for seven days before the experiment. Food and water ad libitum were supplied by following the NIH Guide for the Care and Use of Laboratory Animals. The experimental protocol conducted in this study has been approved by the Institutional Animal Care and Use Committee, China Medical University (104-70-N; 18 December 2014).

### 3.4. Experimental Design of CCl_4_-Induced Hepatotoxicity

Sixty experimental mice were randomly separated into 6 groups. For the control group and the CCl_4_ group, mice were orally administered 1% carboxymethyl cellulose (CMC). For the silymarin group, mice were orally administered silymarin (200 mg/kg in 1% CMC). For the CC_EtOH_ groups, mice were orally administered CC_EtOH_ (20, 100 and 500 mg/kg in 1% CMC). Theses oral administrations were conducted using a feeding tube with 100 μL/10 g Body Weight every day for 9 consecutive weeks. After one week of the administration of the silymarin and experimental drugs, CCl_4_ (40 μL/kg BW, 40% in olive oil) was started to inject intraperitoneally into all mice except for mice in the control group at one hour before the administration of the experimental drugs for every 3.5 days (twice a week) and 8 consecutive weeks. Thus, there were sixteen times of CCl_4_ treatment for the five CCl_4_ treated groups. The control mice received an equivalent volume of olive oil. One week after the last administration of the experimental drugs, the mice were sacrificed under anesthesia and their blood was collected for evaluation of the biochemical parameters (AST, ALT, triglyceride and cholesterol). Their liver tissues were obtained for MDA assay, histological analysis and antioxidant enzymatic activity measurements.

### 3.5. Serum Biochemistry

The serum was obtained as descripted previously [4]. Serum ALT, AST, triglyceride and cholesterol were measured using spectrophotometric diagnostic kits (Roche, Berlin, Germany).

### 3.6. Histological Analysis

Histological Analysis was performed according to the method of the previous report by staining with hematoxylin and eosin [8,11] and with Sirius Red [25], and then observed under light microscopy (Olympus, Tokyo, Japan). For quantitative scoring of the hepatic fibrosis, the values were used according to the published method [14], as the following: none: normal liver, score 0; slight: increase of collagen without formation of septa, score 1; mild: septa do not connect with each other and incomplete septa formation from portal tract to central vein, score 2; moderate: septa interconnecting completely but thin (incomplete cirrhosis), score 3; and remarkable: with thick septa (complete cirrhosis), score 4.

### 3.7. MDA Level as Well as Antioxidant Enzymatic Activity Measurement

MDA level was determined as descripted previously using the thiobarbituric acid reacting substance method [26]. SOD, GPx and GRd enzymatic activities were determined according to the published methods [27,28,29]. MDA, SOD, GPx and GRd assay kits were purchased from Randox Laboratory Ltd. (Antrim, UK).

### 3.8. Statistical Analyses

All data were shown as mean ± SEM. SPSS statistics software program was used to do the statistical data analyses. One-way ANOVA followed by Scheffe’s multiple range test was used to perform the statistical analyses. For the histological analyses, non-parametric Kruskal–Wallis test followed by the Mann–Whitney U-test was used to carry out the statistical analyses. The criterion for statistical significance was *p* < 0.05.

### 3.9. Phytochemical Analysis of CC_EtOH_ by HPLC

The HPLC profile of CC_EtOH_ was determined and compared with the standard (hyperoside and quercetin), which was conducted as descripted previously [20]. Quantification was performed by comparing the sample peak with the corresponding standard compound.

## 4. Conclusions

The present study clearly elucidated that CC_EtOH_ exhibited a hepatoprotective activity against CCl_4_-induced chronic liver injury in mice. The underlying mechanisms were likely the decreasing in MDA level through enhancing the activities of hepatic anti-oxidative enzymes such as GPx, GRd and SOD, and thereby the significant decrease of serum ALT, AST, triglyceride and cholesterol. In addition, fibrosis of liver was significantly reduced by CC_EtOH_. Therefore, *C. campestris* can be developed into pharmacological agents to prevent some liver disorders.

## Figures and Tables

**Table 1:** Quantitative the protective effects of silymarin and CC_EtOH_ on CCl_4_-induced hepatic fibrosis based on histological analyses using Sirius Red staining ^1^.

Group	Histopathologic Score of Liver Fibrosis	
	Observation ^2^	Image (%) ^3^
Normal	0	0.7 ± 0.3
CCl_4_	1.6 ± 0.5	2.9 ± 0.8
Silymarin/CCl_4_	1.4 ± 0.5	1.5 ± 0.5 *
CC_EtOH_ 20 mg/kg/CCl_4_	1.4 ± 0.5	2.1 ± 0.9
CC_EtOH_ 100 mg/kg/CCl_4_	1.2 ± 0.4	2.3 ± 0.7
CC_EtOH_ 500 mg/kg/CCl_4_	1.1 ± 0.5 *	1.9 ± 0.6 *

^1^ Hepatic fibrosis was scored 0–4 according to the method of Ruwart et al. [14] as mentioned in the Materials and Methods; ^2^ The scores were obtained by the following calculation: the sum of the number per grade of affected mice/the total number of examined mice (*n* = 9–10); ^3^ The final Sirius Red positive area (%) was calculated by Image-Plus and was divided the sum of the number per SR positive area (%) of affected mice by the total number of examined mice (*n* = 9–10). * Statistically significant difference between CCl_4_ group and drug-treated groups at *p* < 0.05.

## Supplementary Materials

Supplementary materials can be found at www.mdpi.com/1422-0067/17/12/2056/s1.
