# Supplementary material for: Hepatoprotective Effect of Cuscuta campestris Yunck. Whole Plant on Carbon Tetrachloride Induced Chronic Liver Injury in Mice
Source: Int J Mol Sci. 2016 Dec 7;17(12):2056. doi: 10.3390/ijms17122056 (PMC5187856; doi:10.3390/ijms17122056)
Supplement: Supplementary file 1 [file ijms-17-02056-s001.pdf]

Supplementary Materials: Hepatoprotective Effect of *Cuscuta campestris* Yunck. Whole Plant on Carbon Tetrachloride Induced Chronic Liver Injury in Mice

Wen-Huang Peng, Yi-Wen Chen, Meng-Shiou Lee, Wen-Te Chang, Jen-Chieh Tsai, Ying-Chih Lin and Ming-Kuem Lin

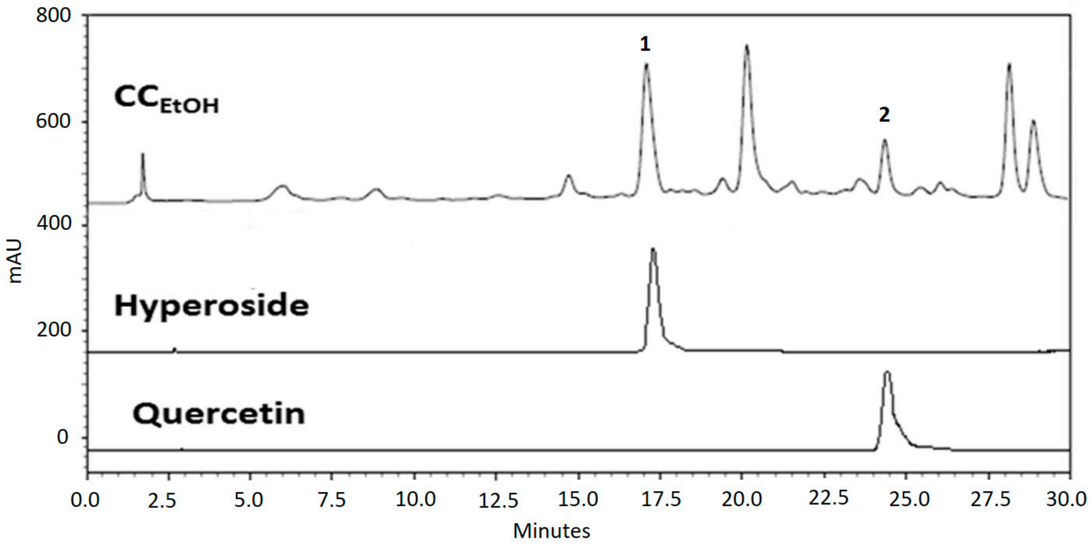

Figure S1. HPLC chromatographs of the ethanol extract of *Cuscuta campestris* whole plant and standards (hyperoside and quercetin). Peaks 1 and 2 are hyperoside and quercetin, respectively.
